# Mongoose (*Herpestes auropunctatus)* May Not Be Reservoir Hosts for *Mycobacterium bovis* in Fiji Despite High Population Density and Direct Contact with Cattle

**DOI:** 10.3390/vetsci6040085

**Published:** 2019-10-24

**Authors:** Philip J. Hayton, Richard J. Whittington, Colin Wakelin, Paul Colville, Aoife Reid, Leo Borja, Jenny-Ann Toribio

**Affiliations:** 1 School of Veterinary Science and Marie Bashir Institute for Infectious Diseases and Biosecurity, Farm Animal Health, University of Sydney, Camden, NSW 2570, Australia; phay9151@uni.sydney.edu.au (P.J.H.); richard.whittington@sydney.edu.au (R.J.W.); 2 Koronivia Research Station, P.O. Box 77 Koronivia, Western Division, Fiji; pmccolville@hotmail.co.uk (P.C.); aoife.reid@hotmail.com (A.R.); borjavets@yahoo.com (L.B.)

**Keywords:** Fiji, mongoose, reservoir host, Mycobacterium bovis, surveillance

## Abstract

The presence of a wildlife reservoir for *Mycobacterium bovis* complicates the eradication of bovine tuberculosis (BTB) from domestic cattle populations. For the BTB eradication program in Fiji, there is concern about the small Indian mongoose (*Herpestes auropunctatus*), which is overabundant and in direct contact with cattle. Consequently, a survey of mongooses trapped on three BTB affected dairy farms led to necropsy of 85 mongooses during January–February 2017. Thirty (35%) mongooses had gross pathological changes including possible granulomas detected at necropsy, and tissues from these animals were taken for histopathological examination. Granulomatous lesions were present in 53% of animals examined histopathologically but acid-fast bacilli were not observed and the majority of lesions in lung and kidney were associated with the nematodes *Pulmostrongylus herpestis* and *Capillaria* sp., respectively. Nevertheless, assuming test sensitivity of 35% for the current study, from this sample of 85 mongooses it can be concluded with 95% confidence that if present in the mongoose population susceptible to trapping, *M. bovis* prevalence was ≤10%. The prevalence of intercurrent lesions raised concerns about gross pathology as a screening test for *M. bovis* infection in mongooses in Fiji, and therefore pathogen detection methods such as bacterial culture and direct tissue PCR are recommended for future surveys. These are needed to completely rule out the mongoose as a reservoir host for *M. bovis* in Fiji.

## 1. Introduction

Bovine tuberculosis (BTB) caused by *Mycobacterium bovis* is a disease of cattle with worldwide distribution that causes paresis, anorexia, wasting, dyspnea, lymphadenomegaly and coughing [1]. In Fiji, BTB has been considered endemic in the cattle population for more than 20 years; it causes significant economic problems due to reduced milk yield and growth rate, the condemnation of milk products and the culling of infected animals under the national brucellosis and tuberculosis eradication program (BTEC) [2]. *M. bovis* is also a concern for human health and its eradication is central to the global tuberculosis (TB) eradication campaign [3,4]. A National Tuberculosis Program has existed to combat TB in Fijian people since 1951 [5]. Recently, the WHO/OIE/FAO released a joint roadmap outlining the future for zoonotic TB management and identified ten priorities, which emphasized improving the scientific evidence, reducing transmission at the animal–human interface and strengthening intersectoral and collaborative approaches to managing zoonotic TB [6]. This study addresses the top priority to improve the scientific evidence base: the surveillance, collection, analysis and reporting on *M. bovis* in wildlife [6].

BTB is present among cattle herds in all Fijian provinces in the Central and Western divisions. In the effort to eradicate this disease the Fijian Ministry of Agriculture currently invests ~1 million USD a year solely for the BTEC program [7]. Despite this control effort, BTB has proven to be difficult to eradicate and therefore an investigation of potential wildlife reservoirs is necessary to more fully understand the epidemiology of BTB in Fiji. In several other countries the eradication of BTB has been problematic due to the potential for disease persistence in wildlife reservoirs, for example badgers in the United Kingdom and brushtailed possums in New Zealand [8,9,10]. Active surveillance is needed to identify candidate reservoir species, and to monitor progress in eradication of *M. bovis* from both the reservoir population and the cattle population [11]. The contribution of wildlife control to cattle BTB eradication has been seen in the surveillance data from New Zealand, which showed declining prevalence of BTB in cattle associated with strict possum control [10]. Thus, confirming or ruling out a role for abundant free-living wildlife that have contact with domestic cattle as reservoirs for *M. bovis* is an important prerequisite for control of BTB.

The small Indian mongoose (*Herpestes auropunctatus*) (family Herpestidae) has features that would make it a potential wildlife reservoir for *M. bovis*. The species already acts as a reservoir for several important zoonotic diseases including bartonella, rabies and leptospirosis, confirming direct or indirect contact with domestic and other wildlife hosts and people [12,13,14]. Mongooses are hardy omnivores with a high reproductive rate, and a large population in Fiji [15]. Anecdotal evidence from Fijian farmers is that large numbers of mongooses are regularly seen scavenging from cattle cull/burial pits located on farms. Whilst the prevalence of *M. bovis* among carcasses in these cull pits is unknown, they could be a point source for *M. bovis* infection for the mongoose population.

Other members of the family Herpestidae are recognized hosts for *Mycobacterium tuberculosis* complex organisms. There are reports of *M. bovis* infection in free-living banded mongoose (*Mungos mungo*) in South Africa [16] and Egyptian mongoose (*Herpestes ichneumon*) in Portugal [17], *M. mungi* in free-living banded mongoose in Botswana and Zimbabwe [18] and *M. suricattae* in free-living meerkats (*Suricata suricatta*) in South Africa [19]. Gross and microscopic lesions typical of tuberculosis were found in these hosts. Consequently, the aim of this study was to use histopathological assessments of gross lesions to screen for tuberculosis in the mongoose population of Fiji, and to determine the feasibility of this approach for future active surveillance in this species.

## 2. Materials and Methods

### 2.1. Survey Design

The purpose of the survey was to assess the occurrence of granulomatous lesions due to mycobacteria in opportunistically trapped mongoose on known BTB-affected farms. The population density of the mongoose had previously been reported as ~10 per hectare [15] and the approximate area of the survey area of Tailevu province is 75,500 hectares. The mongoose population was calculated to be ~755,000 animals [15], which suggested that use of baited traps would be successful.

### 2.2. Animal Ethics Approval

The authors assert that all institutional and national guidelines for the care and use of animals in experimentation were followed in this study. The protocol of this study was reviewed and approved by the Animal Ethics Committee at the University of Sydney (Approval No. 2017/1133).

### 2.3. Trapping and Euthanasia

Based on the BTEC tuberculosis testing records, three dairy farms in Tailevu province, Central division with evidence of persistent high BTB prevalence in cattle were chosen as trapping locations, to increase the likelihood of exposure of the mongoose population to the disease. On each farm, approximately 30 tunnel/cage traps were deployed at equidistant intervals (~20 m) in laneways or scattered around paddocks and sheds on farms for 9 h per day, with checks every 3 h, over 40 days of January–February 2017. Once a triggered trap was retrieved the mongoose was immediately anaesthetized using open halothane inhalation and euthanized with intraperitoneal pentobarbitone. Some dead mongooses were necropsied immediately but most were frozen on the day of trapping and necropsied the following day.

### 2.4. Post-Mortem Examination

For each mongoose, age category was determined based on dentition (young, mature, old), weight was measured using mechanical scales and body length (nose-to-tail and crown-to-rump) was measured with a tape.

Gross evaluation of the integument was performed initially, and any lesions were noted. Then post-mortem proceeded systematically from head to tail [20,21]. The trachea, lung lobes, bronchial lymph nodes and mediastinal lymph nodes were assessed. The liver, stomach, intestines, kidneys, spleen, as well as the hepatic, mesenteric and ileocecal junction lymph nodes were examined visually, by palpation and then dissected. The body wall and urogenital tract were also assessed and the pregnancy status for each female animal was recorded. Visible or palpable gross lesions of any type were recorded, and tissues were collected if tuberculosis was suspected. For histopathology, the lungs and mesenteric lymph nodes were prioritized due to likely routes of infection being inhalation and ingestion, respectively, and to reduce costs. Tissues were fixed in 10× volume of 10% *v*/*v* neutral buffered formalin and transported to the University of Sydney, Australia for further processing. Samples were embedded in paraffin wax, sectioned at 5 microns and stained with hematoxylin and eosin (H&E). Freeze–thaw artefact was not apparent in the majority of histological sections. Following histological assessment, selected tissues containing granulomatous lesions were recut, stained with Ziehl–Neelsen (Z-N) and examined at 1000× magnification under oil immersion to identify acid-fast bacteria.

## 3. Results

### 3.1. Trapping Results and Population Data

Eighty-five wild mongooses (forty-one females and forty-four males) were trapped and euthanized for post-mortem examination. Males (683.6 ± 98.0 g) (mean and standard deviation) were on average approximately 220 g heavier than females (466.4 ± 96.4 g) and 5 cm longer (male 56.5 ± 3.02 cm vs. female 51.48 ± 3.36 cm). Of 25 mature/older females, 20% were pregnant, and the average litter size was two, with the maximum number of pups being three. Mature or older mongooses comprised 76.4% (65/85) of the survey population.

### 3.2. Post-Mortem Findings

Of the eighty-five mongooses that were necropsied, thirty (35.3%) had gross lesions; and various tissues were collected from these for histopathological examination (Table 1 and Table S1). Two mongooses had endometritis at post-mortem; uterine tissues were not collected as the lesions were unlikely to be related to tuberculosis. Fourteen mongoose had gross lesions affecting the lungs, 10 had them in the mesenteric lymph nodes, 5 in the kidney, 4 in the skin, 2 in the liver and 1 each in bronchial lymph node, pre-scapular lymph node, submandibular lymph node, spleen and a few other tissues (Table 1 and Table S1).

### 3.3. Histopathological Findings

**Figure 1:**
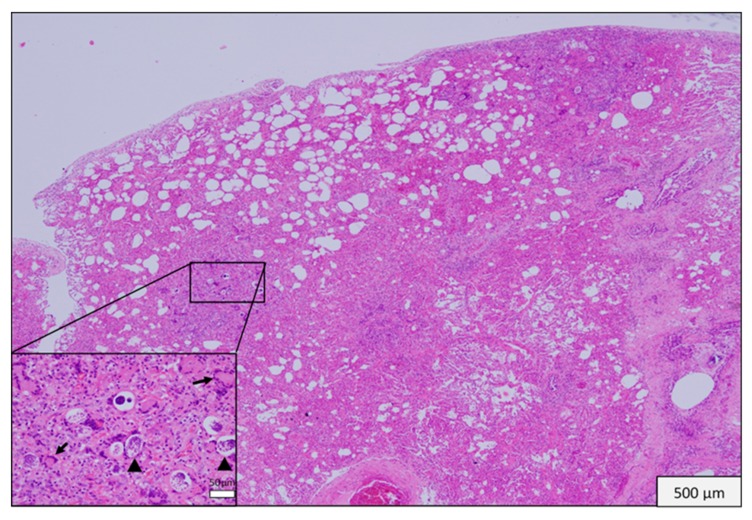
Mongoose 37. Lung. Multifocal granulomatous verminous pneumonia. (Inset) Granulomatous interstitial pneumonia with giant cells (arrows) and nematodes (arrow heads).

**Figure 2:**
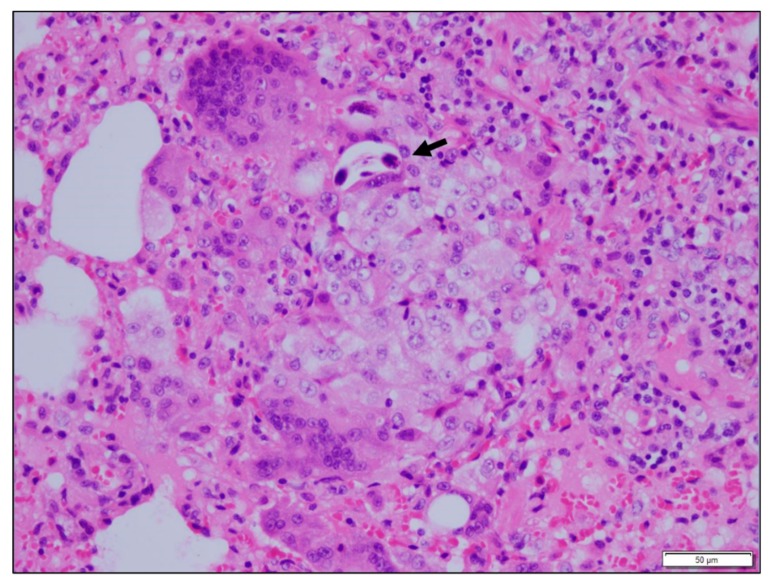
Mongoose 64. Lung. Granuloma with giant cells and epithelioid macrophages. Single nematode in section (arrow).

**Figure 3:**
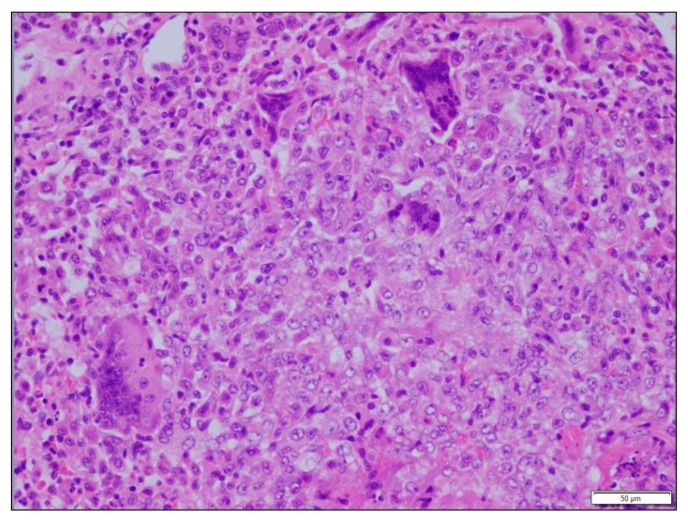
Mongoose 69. Lung. Granuloma with giant cells. No nematodes were visible.

Microscopic lesions were observed in all except five of the tissues collected. In 14 of 30 (46.6%) animals there were changes in lung; there were multifocal microabscesses in one of these mongoose and granulomatous changes in the other 13. The latter ranged in extent and pattern from focal to multifocal bronchial or peribronchial infiltration of macrophages to locally extensive and diffuse interstitial infiltrations and in one there was caseous necrosis with a fibrous capsule. Giant cells were present in lung lesions in seven animals. In 11 of the 14 animals with lung lesions there was mild to moderate congestion and edema (Figure 1). Nematodes were visible in lung sections from 12 of the 13 animals with granulomatous changes but nematodes were not always within or immediately adjacent to these lesions (Figure 2 and Figure 3). The lungworm was morphologically consistent with *Pulmostrongylus herpestis* [22].

**Figure 4:**
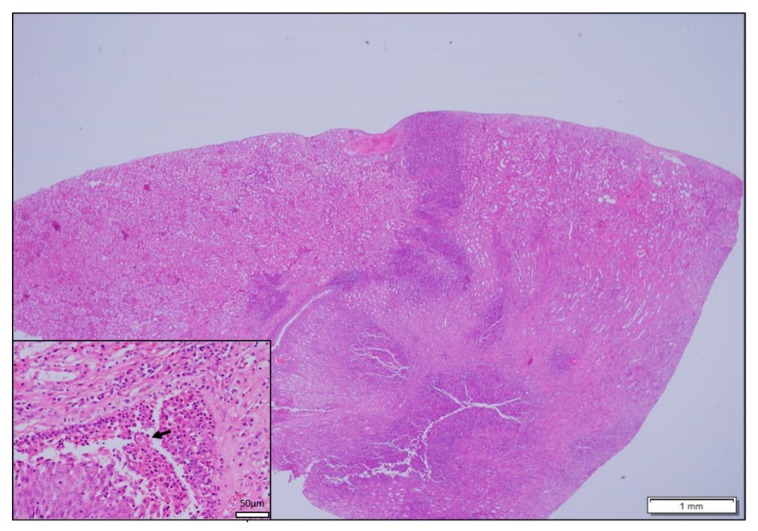
Mongoose 22. Kidney. Chronic active pyelonephritis. (Inset) Mongoose 22. *Capillaria*-type egg (arrow) in lumen of renal pelvis with inflammatory cells.

In the kidney there was mild to severe pyelonephritis affecting 3 of 30 animals (Figure 4). A *Capillaria* egg was observed in the renal pelvis of one mongoose confirming the likely cause of the severe renal lesion as *Capillaria* sp. [23]. The egg was ovoid, had a mamillated outer shell and had a prominent polar cap (Figure 4), consistent with illustrations in Huizinga et al. (1976) [23]. Three mongooses had lesions in both the lung and kidney.

The skin sample taken from one animal contained an abscess and a granuloma with marked infiltration of lymphocytes, giant cells and macrophages and the associated subcutaneous lymph node contained granulomatous infiltrate and was fibrosed, while another animal had pyogranulomatous dermatitis associated with a bite wound. The liver of another mongoose contained multifocal small aggregates of macrophages (microgranulomas). Other morphological diagnoses included myonecrosis and cellulitis, histiocytic hyperplasia/hypertrophy in bronchial or mesenteric lymph nodes and coagulative necrosis in mesenteric lymph node.

Of the 30 mongooses with samples collected at post-mortem, 53.3% (16/30) had caseous necrotic or granulomatous lesions in lung, skin or liver that could suggest infection with *M. bovis* (Table S1). Sections with granulomatous inflammation (except for one due to the presence of fixation artefacts) were examined with a Z-N stain, but no acid-fast bacteria were detected.

## 4. Discussion

*M. bovis* has been reported in a diverse range of species and it is a public health concern in developing countries due to limited resources for control programs. Furthermore, tuberculosis in humans caused by *M. bovis* and *M. tuberculosis* is indistinguishable clinically, radiologically and pathologically [24]. Although the mycobacterium responsible for TB in humans is not always routinely typed, especially in developing countries, it is important to acknowledge the contribution of *M. bovis* which has been estimated to cause 5–10% of human TB cases [25]. In fact, one survey involving isolation and typing showed that 33.9% of all mycobacterial infections in children living along the United States–Mexico border were due to *M. bovis* [26].

*M. bovis* has been shown to persist in wildlife reservoirs in many countries. With regards to the potential for a wildlife species to act as a reservoir it is important to differentiate between a vector host and a spillover host as evidenced by the case of the brushtailed possum (*Trichosurus vulpecula*) in New Zealand [10]. This species of possum is considered to be a vector host, maintaining a persistent source of infection for cattle that are directly exposed to it. Similarly, the badger (*Meles meles*) in England is considered to be a vector host [9], as is the white-tailed deer (*Odocoileus virginianus*) in Michigan, USA [27]. Tuberculosis was identified in wild red deer (*Cervus elaphus*) in New Zealand in 1954 [28] and possums were later shown to be the source of their infection [29]. Wild red deer in New Zealand are spillover hosts, as are feral pigs, stoats, cats, hedgehogs, rabbits, hares, alpacas, goats and sheep, while farmed red deer and wild ferrets can be vector hosts if present at high enough densities [10].

To our knowledge the present study is the first to attempt to identify a wildlife host for *M. bovis* in Fiji. We used gross pathology as a screening test because it is cost effective in a developing country context and has been used successfully to survey wildlife such as brushtailed possums in New Zealand [30] and banded mongoose in Africa [16]. However, even in the definitive host cattle, the sensitivity of post-mortem surveys for tuberculosis has been questioned. One study showed a sensitivity of 31.4% for BTB detection in carcass inspection by Spanish abattoir workers [31] and another study in Ethiopia in 2010 had similar results (30% sensitivity) [32]. In both studies, routine checks failed to identify the majority of positive animals, and this was thought to be due to the low probability (~40%) of an animal presenting to the abattoir with macroscopically detectable lesions (MDL) [30]. Corner (1994) found that cattle with only a single MDL could be identified by a veterinarian 86% of the time by examining three pairs of lymph nodes (mediastinal, medial retropharyngeal and bronchial) and 95% of the time by including the parotid, caudal cervical, superficial inguinal and mesenteric lymph nodes [33]. The post-mortem examinations of mongooses in the present study were conducted by or under the direct supervision of veterinarians and included multiple organ systems because infection may be acquired via different routes.

Bruns et al. (2017) found gross lesions due to *M. bovis* in the lungs and thoracic lymph nodes of banded mongoose, isolated *M. bovis* from abdominal lymph nodes and liver samples and proposed both aerosol and ingestion as potential transmission routes [16]. In other populations of banded mongoose that were infected with *M. mungi,* Alexander et al. (2018) also found gross lesions in the kidney as well as cutaneous lesions [34], and the latter were also found in meerkats with *M. bovis* [35]. Thus, the examination of a wide range of tissues for evidence of *Mycobacterium tuberculosis* complex infection is warranted in the Herpestidae and was undertaken in the present study in Fiji. Although there are no definitive data for mongoose, and regardless of lesion distribution and the veracity of post-mortem examination, the test sensitivity of necropsy for screening for *M. bovis* infection in mongoose would be imperfect. Cross et al. (2000) showed this to be the case in ferrets; nine were infected experimentally per os with *M. bovis*, and only one-third had MDL, primarily in the mesenteric lymph nodes [36]. Therefore, we could assume a test sensitivity of 35% for the current study. In that case, the sample size achieved in this survey means that we can conclude with 95% confidence that if present in the population of mongoose that is susceptible to trapping, *M. bovis* prevalence was ≤ 10% [37]. In surveys of a true reservoir host, the brushtailed possum, that were conducted in New Zealand in the 1970′s using gross pathology, prevalence estimates for *M. bovis* infection were as high as 7.7% in sample sizes > 1400 [30]. Another consideration is that acid-fast bacilli may not be visible in lesions containing viable *M. bovis.* This was shown in South Africa where *M. bovis* was cultured from 10 of 12 banded mongoose; only 2 had gross lesions, and acid-fast bacilli were not visible in these [16]. Hence a larger sample size than that used here, or more sensitive diagnostics, are needed to increase confidence that mongoose in Fiji are not infected with *M. bovis*.

The high incidence of granulomatous inflammatory reactions caused by nematodes (13 of 14 animals with lung samples collected at post-mortem) could have reduced the sensitivity of gross pathological examination to identify tubercular lesions in mongoose in this survey by masking early, small, less widespread or focal lesions due to *M. bovis*. Furthermore, the work and time associated with our strategy of screening by gross pathology, confirming granulomatous lesions by histopathology, and then looking for mycobacteria by acid-fast staining, was noticeably high because of the unexpectedly high prevalence of lesions requiring both microscopic confirmatory tests, which are labor intensive. To address these concerns, future studies should include alternative diagnostic tests such as pathogen detection by microbial culture and direct PCR of tissues [16,17,38]. Due to limited resources in Fiji, these tests were beyond the scope of the present study.

The nematodiasis of the lungs and kidney was severe enough to have significantly compromised normal organ function. This could have increased the likelihood for these animals to enter traps as they shifted from predatory to scavenging behavior with increasing illness [39]. Certainly, systemic disease affects the behavior of another species of mongoose [18]. The nematodes in the lung were consistent with *Pulmostrongylus herpestis* [22] while lesions in the kidney were most probably due to *Capillaria* sp. [23]. As far as we can determine, these two nematodes have not been reported since their original descriptions in Fiji in 1958 (*Pulmostrongylus herpestis*) and 1976 (*Capillaria* sp.) [22,23] and little is known about their distribution in Fiji.

## 5. Conclusions

In conclusion, while evidence of *M. bovis* infection was not found in mongooses, the confounding effects of intercurrent parasitic lesions raised concerns about the utility of gross pathology as a screening test for *M. bovis* infection in this mongoose population in Fiji. Further studies using sensitive pathogen-detection approaches and a larger sample size are needed to rule out the mongoose as a reservoir host for *M. bovis* in Fiji. This will add to the evidence base available to decision makers who determine the target and protocols for the BTEC program in Fiji into the future because *M. bovis* probably cannot be eradicated from cattle in the presence of a wildlife vector host [10]. Furthermore, from a holistic perspective, the ecological impact of parasitic diseases on this population of mongoose merits detailed investigation given the substantial size of this population, the high prevalence of parasitism and the severity of the pathology associated with it, the contact of mongoose with people and livestock and the potential role of the mongoose in disease transmission.

## Figures and Tables

**Table 1:** Prevalence of gross pathological lesions among 85 wild caught mongooses that were trapped on *M. bovis* affected dairy farms in Fiji during January–February 2017. Data for individual mongooses are provided in Table S1.

Organ System	No. of Mongooses (%)	Type of Gross Pathology
Lung	14 (16.5%)	Consolidation, nodules, pallor
Mesenteric lymph node	10 (11.8%)	Lymphadenomegaly
Kidney	5 (5.9%)	Atrophy, pallor, nodules
Skin	4 (4.7%)	Abscess, wound
Liver	2 (4.7%)	Nodules, pallor, mineralization
Submandibular lymph node	1 (1.2%)	Lymphadenomegaly
Bronchial lymph node	1 (1.2%)	Lymphadenomegaly
Prescapular lymph node	1 (1.2%)	Lymphadenomegaly
Spleen	1 (1.2%)	Nodule

## Supplementary Materials

The following are available online at https://www.mdpi.com/2306-7381/6/4/85/s1, Table S1: Summary of tissues examined histologically in each mongoose. c caseous necrosis; g granulomatous inflammation; n nematode or nematode egg; p pyelonephritis.
