# Supplementary material for: Mongoose (Herpestes auropunctatus) May Not Be Reservoir Hosts for Mycobacterium bovis in Fiji Despite High Population Density and Direct Contact with Cattle
Source: Vet Sci. 2019 Oct 24;6(4):85. doi: 10.3390/vetsci6040085 (PMC6958361; doi:10.3390/vetsci6040085)
Supplement: Supplementary file 1 [file vetsci-06-00085-s001.docx]

Table S1. Summary of tissues examined histologically in each mongoose. c caseous necrosis; g granulomatous inflammation; n nematode or nematode egg; p pyelonephritis.

| **Mongoose No.** | **Lung** | | **Bronchial lymph node** | | **Sub-mandibular lymph node** | | **Mesenteric lymph node** | | **Other lymph node** | | **Intestine** | | **Liver** | | **Sub-mandibular salivary gland** | | **Pancreas** | | **Spleen** | | **Kidney** | | **Skin** | | **Subcutaneous lymph nodule** | | **Prescapular lymph node** | | **Totals** |
| --- | --- | --- | --- | --- | --- | --- | --- | --- | --- | --- | --- | --- | --- | --- | --- | --- | --- | --- | --- | --- | --- | --- | --- | --- | --- | --- | --- | --- | --- |
| 2 |  |  | | 1 | |  | |  | |  | |  | | 1 | |  | | 1 | | 1 | |  | |  | |  | | 4 | |
| 5 |  |  | |  | |  | |  | |  | |  | |  | |  | |  | |  | | 1 | |  | |  | | 1 | |
| 11 |  |  | |  | | 1 | |  | |  | |  | |  | |  | |  | |  | |  | |  | |  | | 1 | |
| 17 |  |  | |  | | 1 | |  | |  | |  | |  | |  | |  | |  | |  | |  | |  | | 1 | |
| 19 |  |  | |  | | 1 | |  | |  | |  | |  | |  | |  | |  | |  | |  | |  | | 1 | |
| 21 |  |  | |  | | 1 | |  | | 1 | |  | |  | | 1 | |  | |  | |  | |  | |  | | 3 | |
| 22 | 1 g,n |  | |  | | 1 | | 1 | |  | |  | |  | |  | |  | | 1 p,n | |  | |  | |  | | 4 | |
| 25 | 1 g,n |  | |  | |  | |  | |  | |  | |  | |  | |  | |  | |  | |  | |  | | 1 | |
| 26 |  |  | |  | | 1 | |  | | 1 | |  | |  | |  | |  | |  | |  | |  | |  | | 2 | |
| 30 | 1 n |  | |  | |  | |  | |  | |  | |  | |  | |  | |  | |  | |  | |  | | 1 | |
| 33 |  |  | |  | |  | |  | |  | |  | |  | |  | |  | | 1 | |  | |  | |  | | 1 | |
| 35 | 1 c,g,n |  | |  | |  | |  | |  | |  | |  | |  | |  | | 1 p | |  | |  | |  | | 2 | |
| 36 |  |  | |  | | 1 | |  | | 1 | |  | |  | |  | |  | |  | |  | |  | |  | | 2 | |
| 37 | 1 g,n |  | |  | |  | |  | |  | |  | |  | |  | |  | |  | |  | |  | |  | | 1 | |
| 38 |  |  | |  | |  | |  | |  | |  | |  | |  | |  | |  | | 1 g | |  | |  | | 1 | |
| 40 |  |  | |  | |  | |  | |  | | 1 g | |  | |  | |  | |  | |  | |  | |  | | 1 | |
| 41 | 1 g,n |  | |  | |  | |  | |  | |  | |  | |  | |  | |  | |  | |  | | 1 | | 2 | |
| 43 |  |  | |  | | 1 | |  | | 1 | |  | |  | |  | |  | |  | |  | |  | |  | | 2 | |
| 49 |  |  | |  | |  | |  | |  | | 1 | |  | |  | |  | |  | |  | |  | |  | | 1 | |
| 51 |  |  | |  | |  | |  | |  | |  | |  | |  | |  | |  | | 1 | |  | |  | | 1 | |
| 59 | 2 g,n |  | |  | |  | |  | |  | |  | |  | |  | |  | | 2 p | |  | |  | |  | | 4 | |
| 62 |  |  | |  | |  | |  | |  | |  | |  | |  | |  | |  | | 1 g | | 1 g | |  | | 2 | |
| 64 | 1 g,n |  | |  | |  | |  | |  | |  | |  | |  | |  | |  | |  | |  | |  | | 1 | |
| 67 | 1 g,n |  | |  | |  | |  | |  | |  | |  | |  | |  | |  | |  | |  | |  | | 1 | |
| 69 | 1 g,n |  | |  | |  | |  | |  | |  | |  | |  | |  | |  | |  | |  | |  | | 1 | |
| 73 | 1 g,n |  | |  | |  | |  | |  | |  | |  | |  | |  | |  | |  | |  | |  | | 1 | |
| 74 |  |  | |  | | 1 | |  | |  | |  | |  | |  | |  | |  | |  | |  | |  | | 1 | |
| 79 | 2 c,g,n |  | |  | | 1 | |  | |  | |  | |  | |  | |  | |  | |  | |  | |  | | 3 | |
| 81 | 1 g,n |  | |  | |  | |  | |  | |  | |  | |  | |  | |  | |  | |  | |  | | 1 | |
| 84 | 1 g | 1 | |  | |  | |  | |  | |  | |  | |  | |  | |  | |  | |  | |  | | 2 | |
| No. tissues | 16 | 1 | | 1 | | 10 | | 1 | | 4 | | 2 | | 1 | | 1 | | 1 | | 6 | | 4 | | 1 | | 1 | | 50 | |
| No. animals | 14 | 1 | | 1 | | 10 | | 1 | | 4 | | 2 | | 1 | | 1 | | 1 | | 5 | | 4 | | 1 | | 1 | | 30 | |
